# A Bibliometric Analysis of Literature on the Impact of Diet on Oral Health

**DOI:** 10.7759/cureus.59418

**Published:** 2024-04-30

**Authors:** Namrata Dagli, Mainul Haque, Santosh Kumar

**Affiliations:** 1 Department of Research, School of Dentistry, Karnavati Scientific Research Center, Karnavati University, Gandhinagar, IND; 2 Department of Pharmacology and Therapeutics, National Defence University of Malaysia, Kuala Lumpur, MYS; 3 Department of Periodontology and Implantology, Karnavati School of Dentistry, Karnavati University, Gandhinagar, IND

**Keywords:** dental health, network analysis, thematic analysis, visualization, dietary effects, oral health, diet, review, bibliometric study, scientometric analysis

## Abstract

The complex connection between oral health and dietary habits is fundamental to holistic well-being. Oral health is closely linked to dietary choices, both as a reflection and a factor in overall health. This bibliometric analysis investigates clinical trials published in the PubMed database spanning six decades to understand the landscape of research on the impact of diet on oral health. The analysis reveals a fluctuating yet generally increasing trend over the decades, with significant growth periods interspersed with occasional declines, culminating in a recent slight decrease in research output. With strong collaborative networks, Devizio and Proskin emerge as primary contributors. The University of Sydney has published the maximum number of clinical trials. Thematic clusters highlight diverse research facets, including physiological measurements, socio-economic influences, and biochemical aspects. The evolution in research priorities from experimental studies to longitudinal evaluations underscores the interdisciplinary nature of the field. The analysis of country-wise research output revealed that the United States, Germany, and Australia lead in scientific research output, with notable contributions to the global landscape. While larger countries like China and India are represented, their frequencies are relatively lower, suggesting potential areas for further exploration. Collaboration patterns vary between countries, influencing the global impact of their scientific output. Despite the limitations inherent in database selection, this study integrates bibliometric and thematic analyses to offer comprehensive insights into research trends and priorities.

## Introduction and background

The intricate interrelationship between oral health and dietary habits is a cornerstone of holistic health and well-being. Oral health is intimately intertwined with nutritional choices, serving as both an indicator and a determinant of overall health status [1-4]. Dietary habits play a pivotal role in shaping oral cavity health [5]. The frequency with which food is consumed, along with the types of foods chosen and the consistency of the diet, collectively contribute to either the maintenance or degradation of oral health. For instance, diets high in sugars and carbohydrates have been consistently linked to an increased risk of dental caries [6-9], periodontal diseases [10-13], and oral cancers [14,15]. Conversely, the state of oral health can significantly impact an individual's ability to maintain a nutritious diet [16], affecting aspects such as mastication, taste perception, and nutritional intake. Oral health issues such as periodontal diseases, edentulism, or poorly fitting dental prosthetics can hinder proper mastication, making it difficult to chew and digest certain foods. This can result in dietary restrictions or preferences for softer, easier-to-chew foods, potentially limiting the variety and nutritional quality of one's diet. Additionally, oral health problems can alter taste perception, diminishing the enjoyment and satisfaction derived from eating, which may further influence dietary choices [17,18].

Understanding the dynamic relationship between oral health and dietary habits is essential for devising effective preventive and therapeutic strategies in clinical practice and public health interventions. Through bibliometric analysis, a systematic examination of scholarly literature, we aim to explore the breadth and depth of research dedicated to elucidating this intricate nexus. Bibliometric analysis quantitatively analyzes publications within a specific field or topic. It allows researchers to examine patterns, trends, and relationships within a body of literature, providing insights into the development and impact of research in that area. We have also conducted a thematic analysis to complement this quantitative analysis for a more holistic understanding. Thematic analysis involves identifying and analyzing patterns, themes, and trends within qualitative data. It helps researchers to systematically organize and interpret textual data, uncovering underlying meanings and insights.

This bibliometric analysis aims to explore the scientific literature comprehensively to uncover patterns, trends, and gaps in understanding the relationship between oral health and dietary habits. By synthesizing existing knowledge and identifying emerging research areas, we endeavor to contribute to advancing preventive oral health strategies and promoting holistic approaches to health promotion. Join us as we delve into the dynamic linkage between oral health and dietary habits. We seek to illuminate pathways toward improved oral and systemic health outcomes through informed dietary interventions and holistic health practices.

## Review

Materials and methods

Data Collection

A comprehensive search of the PubMed database was conducted on April 16, 2024, to retrieve relevant English-language clinical trials focusing on the relationship between oral health and diet. The search strategy utilized a combination of Medical Subject Headings (MeSH) terms and keywords related to oral health, diet, and dietary habits. The Boolean operator "NOT" was utilized to exclude animal studies from the research. The following search strategy was employed: (“Oral Health” OR "Dental Health" OR "Dental Caries" OR "Periodontal Diseases" OR "Oral Hygiene" OR “Saliva” OR “Oral Microbiome”) AND ("Diet" OR "Feeding Behavior" OR "Food Habits") AND ("Humans") NOT (Animal).

The filters were applied in PubMed for article type (e.g., clinical trial) and language (e.g., English), and the results were exported to a text file. Then, the text file was manually inspected to ensure the absence of articles other than clinical trials. The process of study selection is depicted in a flow chart generated according to preferred reporting items for systematic reviews and meta-analyses (PRISMA) guidelines [19].

Data Extraction and Analysis

The retrieved data were imported into the Biblioshiny App (RStudio 4.3.1, RStudio, Boston, MA) for bibliometric analysis [20]. Relevant information from each selected clinical trial was extracted, including title, authors, journal, publication year, abstract, study design, intervention(s), outcome measures, and results. In addition, bibliometric analyses, including the identification of leading contributors and collaborators, and thematic analysis were performed using the Biblioshiny App. VOSviewer (version 1.6.20) (Centre for Science and Technology Studies (CWTS), Leiden University, The Netherlands) facilitated the visualization of coauthorship analysis of authors and institutions, as well as co-occurrence networks of keywords, enabling the identification of research themes, trends, and collaborations within the field [21]. Biorender was used to present the study's key findings graphically [22].

The findings of this bibliometric analysis were compiled into a comprehensive report, presenting graphical representations, and interpretive insights to facilitate understanding and dissemination of the results to relevant stakeholders, contributing to the advancement of knowledge in this critical area of healthcare.

Results

**Figure 1:**
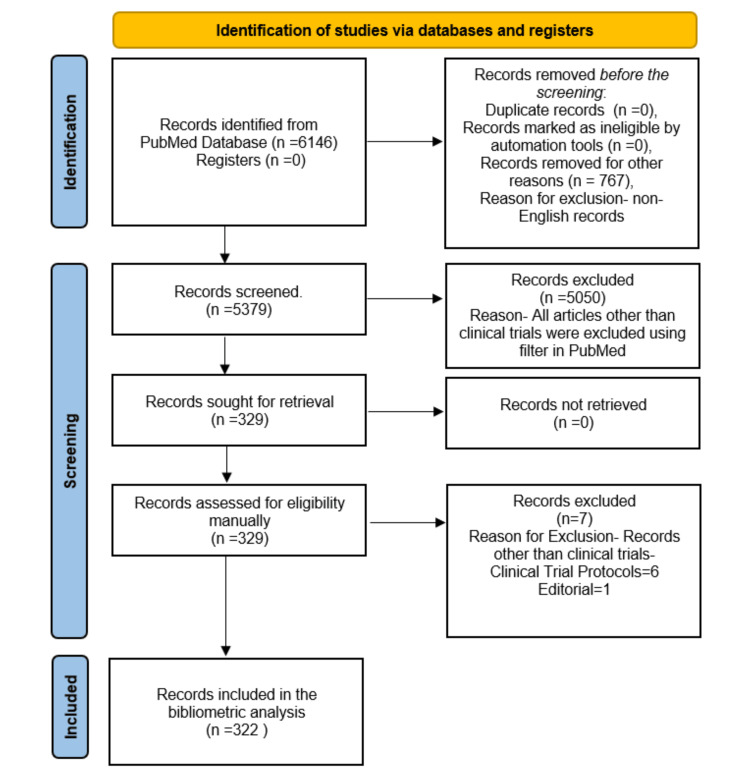
Flow chart of the study selection process for bibliometric analysis of clinical trials on the impact of diet on oral health. Note: This image is the author's own creation.

The initial dataset comprised a total of 6146 articles, which were then filtered based on the English language criteria, resulting in 5379 articles. Among these were 655 reviews, 329 clinical trials, 110 case reports, 51 comments, 40 editorials, and one book. After manual inspection, which likely involved assessing the relevance and quality of the articles, six protocols, and one editorial were excluded from further analysis. Finally, a subset of 322 clinical trials was selected for detailed analysis, suggesting a rigorous process to ensure the inclusion of relevant studies meeting specific criteria for the research focus (Figure 1).

Publishing Trends

**Figure 2:**
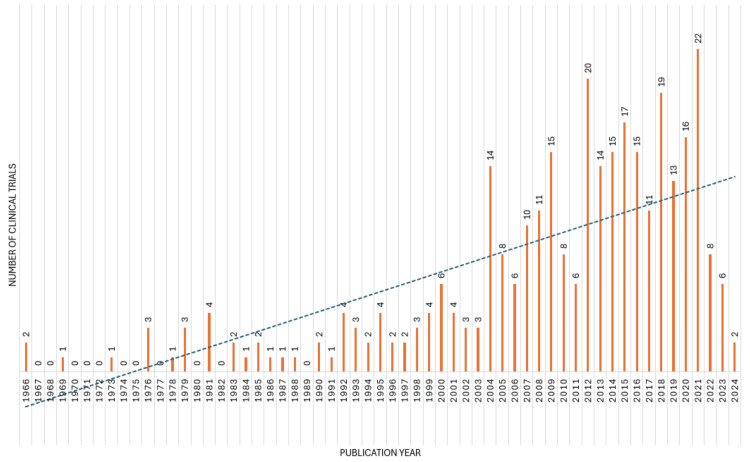
Annual scientific publications of clinical trials on the impact of diet on oral health. Note: This image is the author's own creation.

Over the past six decades, the landscape of clinical trials focusing on the impact of diet on oral health has shown significant fluctuations. In the initial years, from 1966 to the early 1980s, the number of publications remained quite low, with occasional spikes. From 1976, there was a small surge, peaking at four articles in 1981. However, this was followed by a decline, with intermittent increases in the following years. The trend began to change notably in the late 1990s, with a steady increase in publications, reaching a peak of 14 articles in 2004. This period witnessed substantial growth, with percentages often in the double digits. The surge continued into the mid-2010s, marked by peaks in 2012 (20 articles) and 2015 (17 articles). The last five years have shown a somewhat declining trend, with a slight decrease in the number of publications. This is particularly evident in the years 2021 and 2022, which saw 22 and eight articles, respectively. The year 2023 shows only six articles published, marking one of the lowest points in recent years. Overall, the data suggests an increasing interest in the impact of diet on oral health, with peaks and troughs reflecting shifts in research focus and scientific priorities. While there have been fluctuations, the general trend has been one of growth, albeit with recent indications of a potential slowdown or stabilization in research output (Figure 2).

Most Relevant Authors

**Figure 3:**
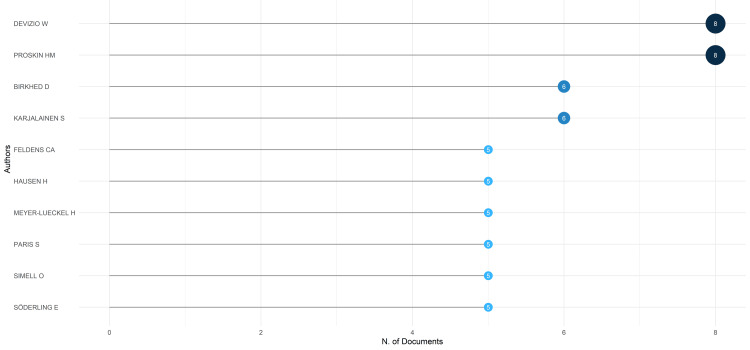
The most relevant authors are based on the number of published clinical trials in PubMed on the impact of diet on oral health. Note: This image is the author's own creation.

Based on our analysis, Devizio and Proskin emerged as the primary contributors, considering the number of publications. These two authors individually authored eight clinical trials each, collectively comprising approximately 27.6 publications among the top ten most contributing authors (Figure 3). Together, the ten authors accounted for nearly 18% of the total clinical trials published in PubMed.

Coauthorship Analysis of Authors

**Figure 4:**
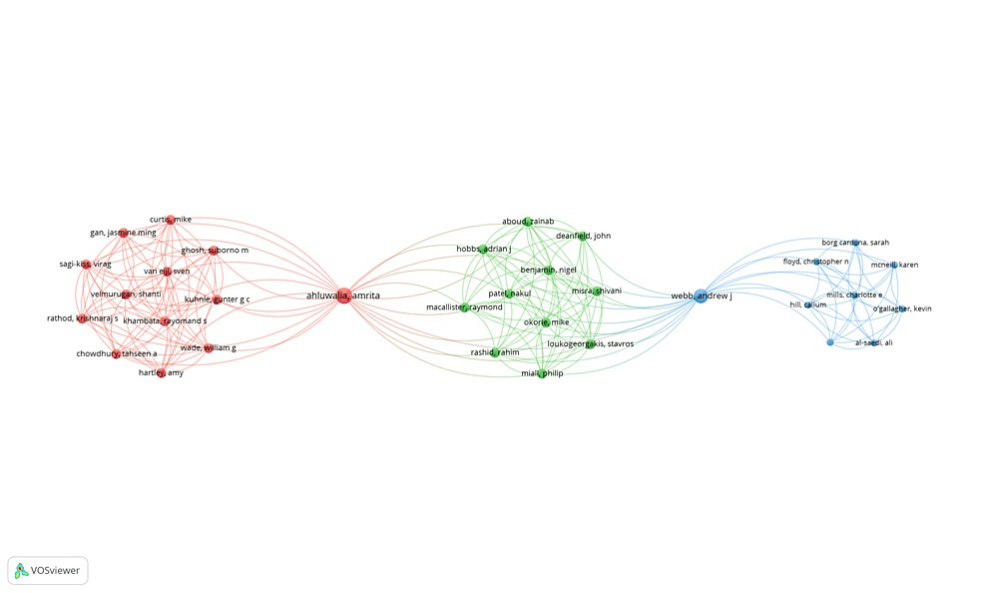
Coauthorship analysis of authors—network visualization of the largest set of connected authors. The nodes represent the authors, and the lines represent the link between them. The size of nodes is directly proportional to the number of links. (Weight: Links, cluster 1 is red, cluster 2 is green, and cluster 3 is blue). Note: This image is the author's own creation.

A total of 1622 authors were identified, and all were included in the analysis, with total link strength (TLS) values calculated by VOSviewer. However, it's important to note that not all 1622 authors were connected. The largest set of connected authors consisted of 33 authors, and the collaboration network of these authors is depicted in Figure 4. Within this network, the authors are distributed across three clusters, with 192 links identified. Cluster 1 is represented in red, cluster 2 in green, and cluster 3 in blue. The size of the nodes in the figure is directly proportional to the number of links. Notably, authors Devizio and Proskin exhibited the highest TLS values of 41, with eight publications each.

Most Relevant Institutions

**Figure 5:**
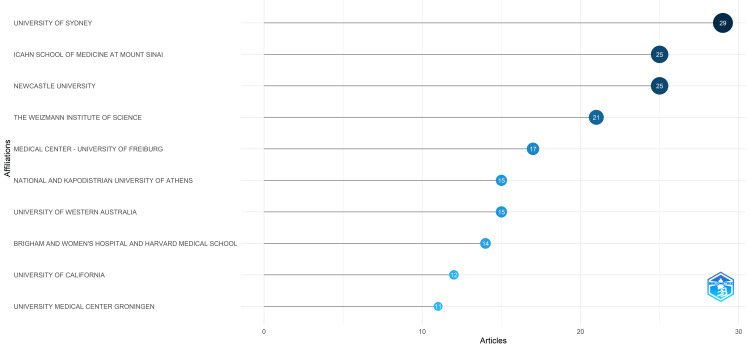
The most relevant institutions based on the number of published clinical trials in PubMed on the impact of diet on oral health. Note: This image is the author's own creation.

Several institutions emerge as notable contributors in our analysis by the Biblioshiny App. The University of Sydney, based in Australia, leads the list with 29 articles, representing approximately 9% of the total publications included in the analysis. The Icahn School of Medicine at Mount Sinai and Newcastle University are closely followed, with 25 articles each. The former, located in the United States, and the latter, based in the United Kingdom, contribute approximately 7.76% each to the total publications. These institutions demonstrate substantial dedication to understanding the relationship between diet and oral health. The Weizmann Institute of Science in Israel follows with 21 articles, accounting for approximately 6.52% of the publications. Despite not being a medical institution, its multidisciplinary approach likely enriches the research landscape in this field. Furthermore, the Medical Center-University of Freiburg in Germany and the National and Kapodistrian University of Athens in Greece each contribute 17 and 15 articles, respectively, making up approximately 5.27% and 4.66% of the total publications. These international contributions highlight a diverse and global interest in studying the impact of diet on oral health. Overall, the collective efforts of these institutions signify a rich body of research that informs both clinical practice and public health interventions worldwide (Figure 5).

Coauthorship Analysis of Organizations

**Figure 6:**
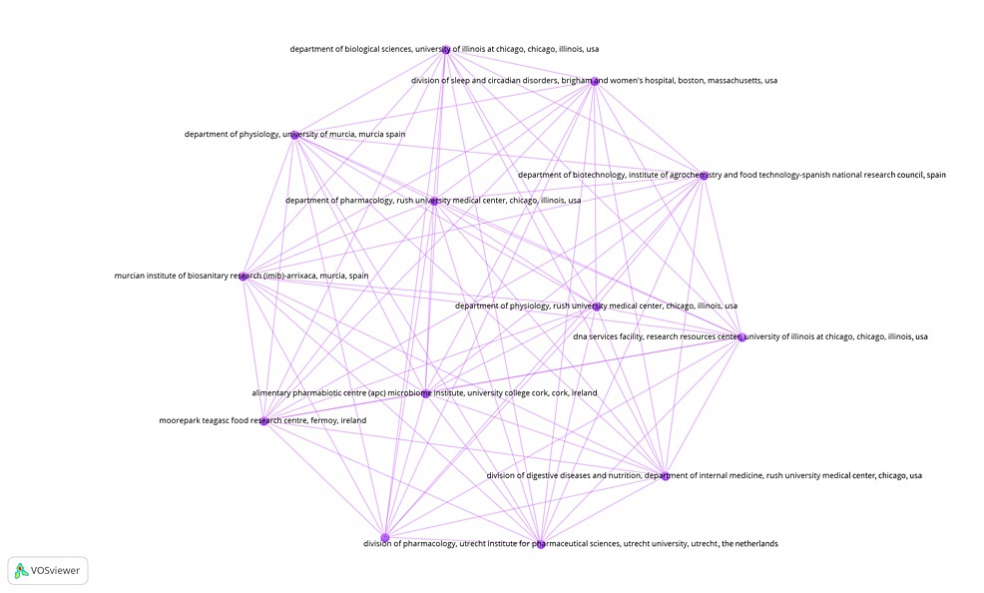
Coauthorship analysis of institutions-network visualization of the largest set of connected authors. The nodes represent the authors, and the lines represent the link between them. The size of the nodes is directly proportional to the number of links (Weight: Links). Note: This image is the author's own creation.

A total of 636 organizations were identified, of which six met the threshold of having a minimum of two published clinical trials. Each of the 636 organizations was included in the coauthorship analysis conducted by VOSviewer, and TLS was calculated. It was observed that some institutions were not interconnected. The largest group of connected institutions consisted of 13 institutions, as depicted in Figure 6. All 13 items were grouped under one cluster with 78 links. Also, these institutions had the highest TLS value of 12 and one publication each.

Most Relevant Journals

**Figure 7:**
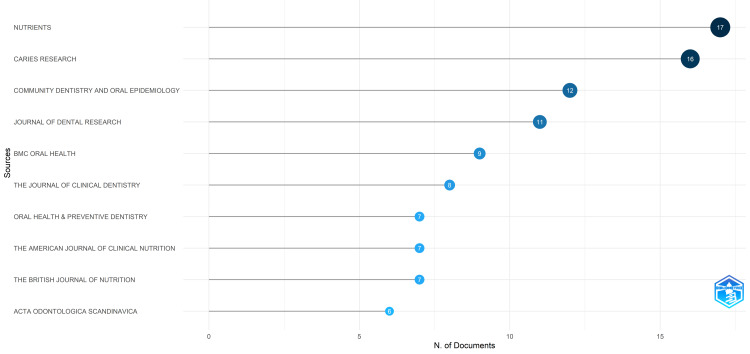
The most relevant journals are based on the number of published clinical trials in PubMed on the impact of diet on oral health. Note: This image is the author's own creation.

Nutrients emerged as the foremost source, as indicated by the volume of published clinical trials, trailed by Caries Research. Together, these two journals contributed to 33 clinical trials, representing roughly 10.25% of the publications in PubMed. While the top ten journals contributed to almost 31% of these publications (Figure 7).

Co-occurrence Analysis of Keywords

**Figure 8:**
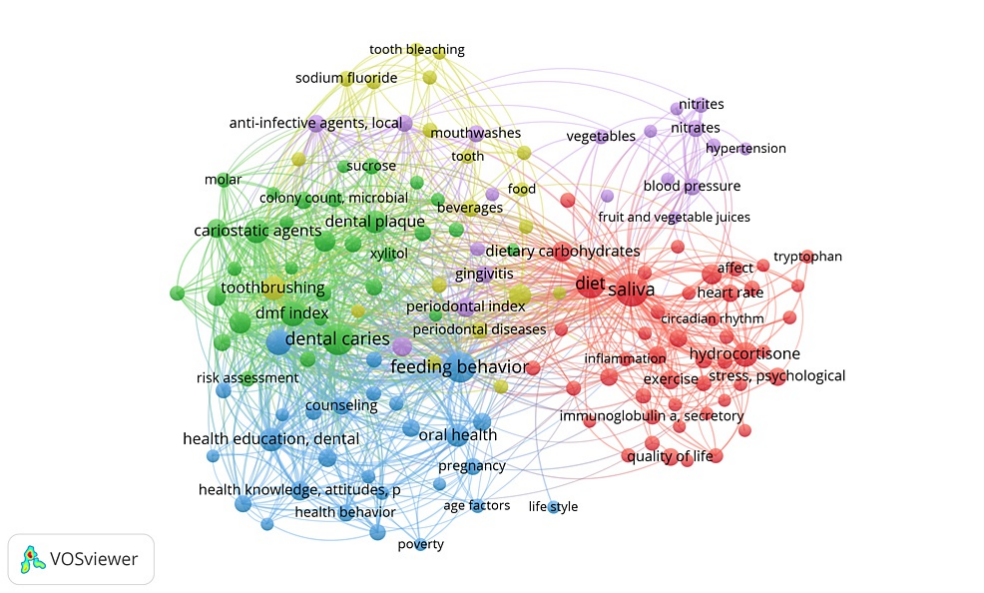
Network visualization of co-occurrence analysis of the most frequently used keywords in clinical trials on the impact of diet on oral health. The size of the nodes represents the keywords, and the connecting lines represent the links between them. (Weights: occurrences; cluster 1 is red, cluster 2 is green, cluster 3 is blue, cluster 4 is yellow, and cluster 5 is purple). Note: This image is the author's own creation.

A total of 1155 MeSH keywords were identified, of which only 182 met the criteria of a minimum occurrence of 5. The total strength of co-occurrence links with other keywords was calculated for each of these 182 keywords using VOSviewer. According to the analysis, keywords such as "human," "female," "male," and "adult" showed the highest total link strength (TLS). However, as these keywords primarily describe the study population without providing subject-specific information, they were excluded along with other nonspecific words when generating the network visualization (Figure 8). The resulting visualization included 145 keywords spread across five clusters, with 2916 links and a TLS of 7541. After removing the nonspecific terms, the keywords with the highest TLS identified are saliva, dental caries, feeding behavior, diet, and oral hygiene. Each cluster is depicted in a different color: cluster 1 in red, cluster 2 in green, cluster 3 in blue, cluster 4 in yellow, and cluster 5 in purple. The keywords in each cluster are detailed in Table 1.

**Table 1:** Keywords in clusters were identified in the co-occurrence analysis of keywords.

Clusters	Keywords
Cluster 1 (60 items)	adult, affect, aged, aged 80 and over, amino acids, anthropometry, athletic performance, biomarkers, blood glucose, body composition, body mass index, body weight, c-reactive protein, caffeine, caloric restriction, chewing gum, circadian rhythm, cross-over studies, cytokines, diet, diet records, dietary carbohydrates, dietary proteins, dietary supplements, drug dose-response relations, double-blind method, eating, energy intake, energy metabolism, exercise, fatty acids, female, healthy volunteers, heart rate, humans, hydrocortisone, immunoglobulin a, immunoglobulin a secretory, inflammation, mastication, lifestyle, male, nutritional physiological, nutritional status, obesity, overweight, physical endurance, pilot projects, quality of life, saliva, salivation, stress psychological, taste, time factor, tryptophan, weight loss, xerostomia, young adult
Cluster 2 (58 items)	Age factors, attitude to health, bacterial load, brazil, breastfeeding, caregivers, cariostatic agents, chi-square distribution, child, child behavior, child preschool, cohort studies, counseling, dental care, dental caries, dental caries susceptibility, dental plaque index, dental prophylaxis, permanent dental restorations, dietary sources, DMF (decayed missing filled) index, educational status, feeding behavior, fluorides, topical fluorides, follow-up studies, health behavior, health education, dental health education, health knowledge, attitude, practice, health promotion, incidence, infant, newborn infant, longitudinal studies, molar, mothers, motivation, motivational interviewing, oral health, oral hygiene, parents, patient education as topic, pit and fissure sealants, poverty, pregnancy, prenatal care, program evaluation, prospective studies, research design, risk assessment, risk factors, school dentistry, social class, teaching, deciduous tooth, toothbrushing, toothpaste
Cluster 3 (31 items)	analysis of variance, carbonated beverages, cariogenic agents, clinical trial as a topic, microbial colony count, dental enamel, dental plaque, dentin, cariogenic diet, disease progression, Finland, fructose, gingivitis, hydrogen ion concentration, Iran, lactobacillus, mouth, patient compliance, periodontal index, placebos, probiotics, regression analysis, secretory rate, single-blind method, smoking, nonparametric statistics, Streptococcus mutans, sucrose, sweetening agents, tooth demineralization, xylitol
Cluster 4 (18 items)	adolescent, beverages, case-control studies, cross-sectional studies, dental devices home care, dentifrices, drug combinations, food, oxidants, pediatric obesity, periodontal diseases, prevalence, sodium fluoride, Sweden, tooth, tooth bleaching, tooth discoloration, treatment outcome
Cluster 5 (15 items)	local anti-infective agents, bacteria, beta vulgaris, blood pressure, chlorhexidine, fruit, fruit and vegetable juices, hypertension, microbiota, mouthwashes, nitrates, reference values, vegetables

The keywords in cluster 1 indicate that the research seems to focus on the impact of diet on oral health in adults, particularly about various factors such as age, diet composition (including amino acids, dietary carbohydrates, dietary proteins, fatty acids, etc.), biomarkers (such as blood glucose, c-reactive protein, cytokines, immunoglobulin A), lifestyle factors (such as exercise, stress, caffeine intake), physiological measurements (body composition, body mass index, energy metabolism), and psychological factors (quality of life, stress). It might also investigate how factors like age, gender, or lifestyle habits influence these relationships. Additionally, the inclusion of terms like "saliva" and "xerostomia" suggests a focus on the physiological aspects of oral health, such as saliva production and its role in protecting the teeth and gums.

The keywords in cluster 2 suggest a research focus on various factors such as age, attitudes toward health, bacterial load, cultural factors (e.g., Brazil), breastfeeding, caregivers' influence, cariostatic agents, statistical methods (e.g., chi-square distribution), child-related factors including behavior and preschool-aged children, longitudinal studies, dental care and hygiene practices, dental caries susceptibility and its assessment (e.g., DMF index), educational status and its influence, feeding behavior and dietary sources, fluorides and their role in oral health (both topical and dietary sources), health education and promotion strategies, incidence and risk assessment of dental issues, maternal factors including prenatal care and breastfeeding, program evaluation and prospective studies, socio-economic factors such as poverty and social class, teaching methods related to oral hygiene and health promotion, tooth development, toothbrushing techniques and use of toothpaste, motivational interviewing as a counseling approach, and the use of dental interventions such as pit and fissure sealants. Research in this area might involve various study designs, including cohort studies, follow-up studies, cross-sectional studies, and prospective studies. It would likely encompass a multidisciplinary approach, drawing from fields such as dentistry, public health, psychology, and nutrition, among others. The research could aim to understand the complex interactions between dietary factors, oral hygiene practices, socio-economic determinants, and individual behaviors influencing oral health outcomes across different age groups and cultural contexts.

The keywords in cluster 3 indicate the nuanced effects of dietary components, such as carbonated beverages, sugars like sucrose and fructose, and sweetening agents, on oral health outcomes studied through analysis of variance (ANOVA) and regression analysis. Researchers likely aim to discern clinical trials, employing methods like the single-blind technique and placebos, which may be utilized to evaluate interventions aimed at improving oral health and assessing patient compliance. Microbial ecology within the oral cavity, including factors like microbial colony count, dental plaque composition, and the presence of bacteria like *Streptococcus mutans *and Lactobacillus, likely forms a key area of investigation. Additionally, the impact of diet on dental structures, such as enamel and dentin, and processes like tooth demineralization, as well as factors like saliva secretory rate and hydrogen ion concentration, may be explored. Comparative studies between populations in Finland and Iran could shed light on the influence of cultural and dietary differences on oral health outcomes. Furthermore, considerations of smoking as a potential confounding factor and the use of nonparametric statistics suggest a comprehensive approach to understanding the complex relationship between diet and oral health, with implications for disease prevention and management strategies. 

The keywords in cluster 4 suggest that research explores the multifaceted relationship between diet and oral health, with a particular focus on adolescents. Through case-control and cross-sectional studies, it delves into the impact of various dietary factors, including beverages, food choices, and oxidants, on prevalent oral health issues such as periodontal diseases and tooth discoloration. Additionally, it examines the influence of dental devices and dentifrices in-home care routines and assesses treatment outcomes regarding dietary habits and drug combinations. The prevalence of pediatric obesity is considered alongside dietary patterns, while the role of fluoride intake, notably in sodium fluoride, is analyzed for its effects on oral health outcomes. Geographically, the research could originate from Sweden. Overall, the investigation aims to offer insights into preventive strategies and treatment approaches in adolescents.

The keywords in cluster 5 suggest a comprehensive exploration of the efficacy of various local anti-infective agents and mouthwashes in preventing oral infections and maintaining oral hygiene. Additionally, investigations would focus on understanding the role of bacteria and microbiota in oral health, including how dietary choices influence the composition and balance of oral microbiota. Studies would also examine the impact of fruit, vegetable, and juice consumption on oral health. Specific dietary components like beta vulgaris and nitrates would be scrutinized for their potential benefits or risks to oral health. Furthermore, the interplay between diet, blood pressure regulation, and hypertension will be explored to elucidate its implications for oral health outcomes. Collectively, the research endeavors aim to establish evidence-based guidelines and reference values for dietary intake, informing effective strategies to promote optimal oral health through dietary interventions.

Thematic Map

**Figure 9:**
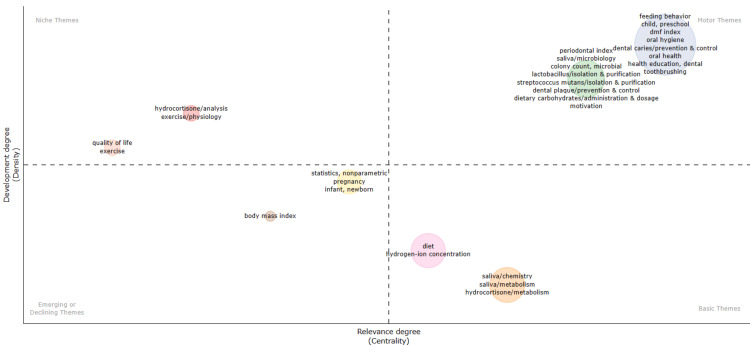
Thematic map of the most frequently used keywords in clinical trials on the impact of diet on oral health in PubMed. The analysis is done using the Biblioshiny App. Note: This image is the author's own creation.

The thematic mapping reveals distinct clusters of themes, each indicative of varying levels of relevance and development (Figure 9). Well-established niche themes such as hydrocortisone analysis, exercise physiology, and quality of life represent specialized areas of study within the broader context of oral health research. Conversely, basic terms like diet, hydrogen ion concentration, and salivary chemistry, while pertinent to the subject, exhibit comparatively less development. This suggests that there is significant scope for further exploration, research, and development in these areas. The motor themes, comprising a comprehensive array of well-developed and relevant concepts such as periodontal index, feeding behavior, dental caries prevention, dental plaque, the microbiology of saliva, isolation of *S. mutans* and Lactobacillus, dietary carbohydrates, and oral hygiene, serve as the driving force behind the advancement of research in this field. The motor themes imply that the research landscape is characterized by active exploration, advancement, and emphasis on practical application in the field of oral health and diet. Meanwhile, emerging or declining themes such as body mass index, nonparametric statistics, and studies on pregnancy and newborn infants suggest shifting research priorities over time. By delineating these thematic patterns, researchers can navigate the landscape of oral health research, identifying areas ripe for further exploration and discerning trends in scholarly inquiry.

Analysis of Topic Trends

**Figure 10:**
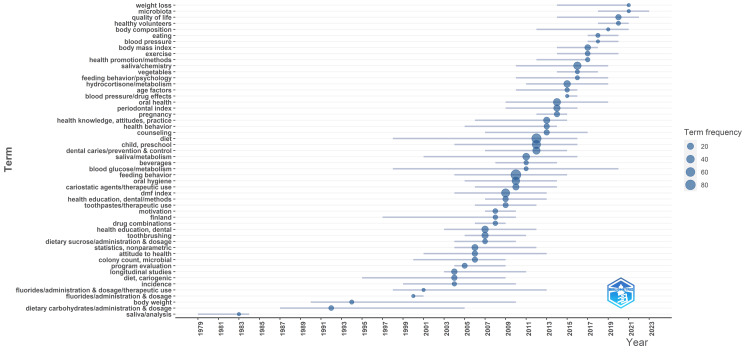
Analysis of topic trends of clinical trials on the impact of diet on oral health in PubMed. Note: This image is the author's own creation.

Over the decades, research on the impact of diet on oral health has evolved significantly, reflecting changing scientific priorities and methodologies. In the early stages, particularly during the 1970s and 1980s, there was a notable emphasis on clinical trials and saliva analysis, highlighting efforts to understand oral health from an experimental perspective. As the field progressed into the late 1980s and early 1990s, attention shifted towards dietary carbohydrates and body weight, indicating a growing recognition of the role of diet in oral health outcomes, as well as its connection to broader health concerns. The turn of the millennium saw a surge in longitudinal studies, demonstrating a shift towards investigating the long-term effects of diet on oral health, alongside increased research on cariogenic diets, reflecting efforts to identify specific dietary factors contributing to dental caries. Throughout the 2000s and 2010s, there was a notable emphasis on fluorides and behavioral factors such as toothbrushing, motivation, and feeding behavior, highlighting a growing understanding of the complex interplay between individual behaviors and oral health outcomes. The term hydrocortisone metabolism is the most prevalent term in 2015, indicating a study on the interrelationship of stress, diet, and oral health. Moreover, the latter half of the 2010s and into the 2020s witnessed a deepening exploration of biological mechanisms and overall well-being related to oral health and dietary habits, with increasing research interest in topics such as saliva chemistry, microbiota, overall well-being, and quality of life. This trajectory underscores a holistic approach to understanding the relationship between diet and oral health, encompassing experimental, behavioral, demographic, and biological dimensions (Figure 10).

Thematic Evolution

**Figure 11:**
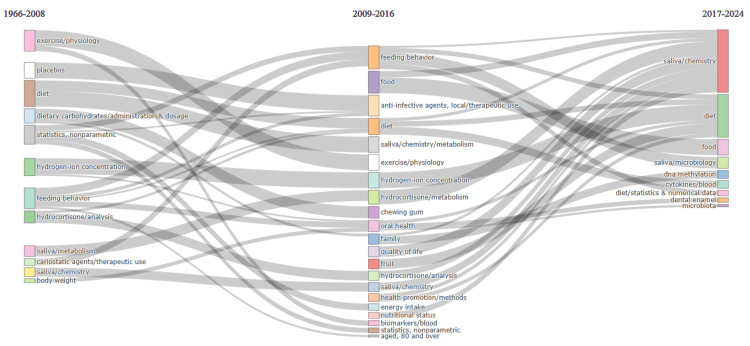
Thematic evolution analysis in clinical trials on the impact of diet on oral health in the PubMed database. The analysis is done using the Biblioshiny App. Note: This image is the author's own creation.

The data analysis reveals a comprehensive evolution in research concerning the impact of diet on oral health. Over time, there has been a consistent focus on understanding the intricate relationship between dietary habits and oral health outcomes (Figure 11). Initial research emphasized specific dietary factors like carbohydrates and sucrose in the context of dental caries prevention. However, in later years, the scope expanded to encompass broader dietary patterns, including factors such as body mass index, energy intake, and waist circumference, indicating a more holistic approach to studying dietary impact. Alongside dietary factors, research also delved into intervention strategies, with a particular emphasis on toothbrushing methods, fluoride administration, and the therapeutic use of substances like xylitol. Notably, interdisciplinary perspectives emerged, with studies exploring the interaction between dietary factors and physiological processes in the oral cavity. Furthermore, there was a shift towards longitudinal studies and program evaluations to assess the long-term effectiveness of dietary interventions on oral health outcomes. Importantly, social and behavioral determinants were also recognized as critical factors shaping dietary habits and, consequently, oral health. This multifaceted understanding underscores the need for comprehensive approaches that address dietary factors alongside socio-behavioral determinants to promote optimal oral health and overall well-being.

Scientific Production of Countries

**Figure 12:**
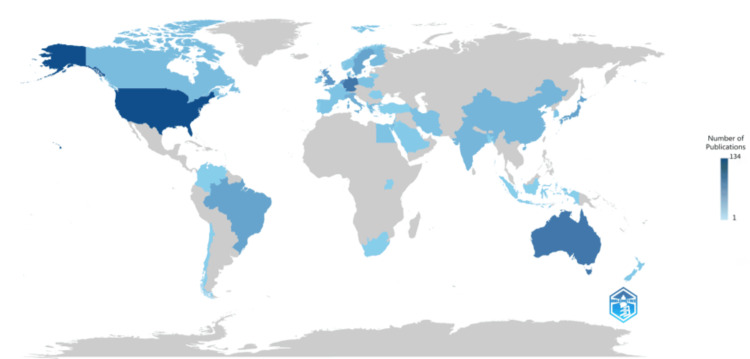
Scientific production of countries. The shade of blue color represents the frequency of publications. The gray color represents no publication. Note: This image is the author's own creation.

The dataset presents a comprehensive overview of the distribution of scientific research output across numerous countries (Figure 12). Notably, the United States emerges as the leading contributor with 134 articles, underscoring its prominent role in global scientific research. Germany and Australia follow closely behind, with 91 and 89 articles, respectively, demonstrating their significant contributions to the scientific community. The United Kingdom, Netherlands, and Japan also feature prominently in the rankings, each with a substantial number of articles attributed to them. Interestingly, while larger countries like China and India are represented in the dataset, their frequencies are relatively lower compared to their population sizes and research capacities. This suggests potential areas for further exploration into the dynamics of scientific output within these countries. Additionally, the presence of articles from smaller nations like Albania, Bangladesh, and Uganda highlights the global reach of scientific inquiry, emphasizing the importance of inclusivity and collaboration in advancing knowledge across borders. Overall, this dataset provides valuable insights into the geographic distribution of scientific research activity and underscores the diversity and richness of the global scientific landscape.

**Figure 13:**
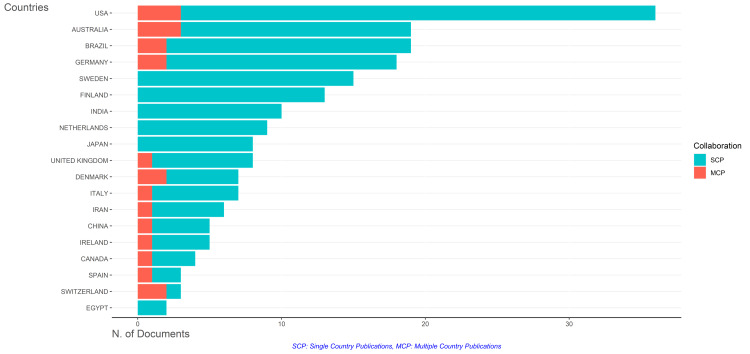
The frequency of collaboration of corresponding authors’ countries in research on the impact of diet on oral health. The analysis is done using the Biblioshiny App. Note: This image is the author's own creation.

The dataset provides insights into scientific article publications across various countries, focusing on the number of articles and collaboration patterns. Notably, the United States emerges as the most prolific contributor, followed closely by Australia and Brazil. Some countries exhibit a strong tradition of international collaboration, as evidenced by a significant number of articles involving researchers from multiple nations. The USA and Australia have the highest number of multiple-country publications, followed by Brazil, Germany, Denmark, and Switzerland. Conversely, other countries demonstrate a preference for single-country publications, such as Sweden, Finland, India, Netherlands, Japan, and Egypt. These findings underscore the diverse strategies employed by different nations in scientific research dissemination, with potential implications for the global impact and visibility of their scientific output. Additionally, it's worth noting that the analysis includes countries of corresponding authors (Figure 13).

Discussion

The analysis of clinical trial publications on the impact of diet on oral health over the past six decades reveals fluctuating trends with notable peaks in 2021, followed by a recent decline, suggesting a general increasing interest in the topic but with indications of potential stabilization in research output. The maximum number of publications in 2021 could be attributed to several factors. One possibility is that there was a significant breakthrough or advancement in research related to the impact of diet on oral health, prompting a surge in clinical trials and publications. Another reason could be an increased awareness of the importance of dietary factors in oral health, leading to heightened interest and funding for related research projects. As for the decline in publications during recent years, particularly after 2021, several factors could be at play. One potential reason is that researchers may have shifted their focus to other emerging areas within oral health or broader areas of nutrition and health, diverting resources and attention away from studies specifically examining the impact of diet on oral health. Additionally, changes in funding priorities or limitations in resources could have constrained the ability of researchers to conduct large-scale clinical trials in this field. Moreover, some of the key questions or hypotheses regarding the relationship between diet and oral health may not have been sufficiently explored, leading to a natural decline in research activity as fewer novel avenues remain to be investigated. 

Devizio and Proskin emerge as primary contributors based on the number of published clinical trials. In addition, coauthorship analysis unveils a complex network of collaboration among authors, with Devizio and Proskin exhibiting strong ties with other researchers. Moreover, institutions such as the University of Sydney, Icahn School of Medicine at Mount Sinai, and Newcastle University stand out for their substantial contributions, indicating a global interest in understanding the interplay between diet and oral health. Collaborative efforts among these institutions contribute to a rich body of research that informs clinical practice and public health interventions worldwide. Nutrients emerged as the foremost source, based on the volume of published clinical trials, trailed by Caries Research.

The identified thematic clusters shed light on the diverse facets of research within this field. Cluster 1 suggests focusing on diet composition, biomarkers, physiological measurements, and psychological factors, particularly in adults. Cluster 2 suggests focusing on cultural factors, dental caries assessment, and cariostatic agents influencing oral health across different age groups and socio-economic backgrounds. Cluster 3 indicates a focus on probiotics and prevention of tooth demineralization. Cluster 4 indicates the prevalence of oral diseases, treatment outcomes, and effects of beverages among adolescents. Cluster 5 indicates a research focus on microbial ecology and the efficacy of dietary interventions in preventing oral infections. These clusters underscore the importance of considering not only dietary factors but also demographic, behavioral, and lifestyle aspects in understanding oral health outcomes. The thematic map that the research landscape is characterized by active exploration, advancement, and emphasis on practical application in the field with few areas that need further development, such as saliva chemistry, saliva metabolism, hydrocortisone metabolism, and hydrogen ion concentration. Research into hydrogen ion concentration in diet and oral health examines how dietary factors influence the pH balance of saliva and oral fluids, and how variations in pH may impact dental health outcomes such as tooth erosion, enamel demineralization, and oral microbiome balance [23]. In addition, the change in the salivary pH toward acidity has been associated with anxiety [24]. Research into hydrocortisone metabolism in the context of diet and oral health investigates how dietary factors influence the body's production, regulation, and metabolism of hydrocortisone, a key steroid hormone involved in stress response and inflammation. Dietary nutrients, gut microbiota composition, inflammatory responses, and stress levels all play roles in modulating cortisol levels and signaling pathways [25-27]. Dysregulation of hydrocortisone metabolism due to poor dietary habits or chronic stress can impact oral health outcomes by influencing inflammatory conditions, immune responses, and wound-healing processes in the oral cavity. Furthermore, eating disorders are also associated with psychological stress, indicating the complex interrelationship of stress, diet, and oral health [28-30]. Understanding these interactions is crucial for developing effective dietary interventions and preventive strategies to promote oral health and manage oral diseases.

Analysis of topic trends reveals a dynamic evolution in research priorities over time. From early emphasis on experimental studies and saliva analysis to contemporary investigations into microbiota and quality of life, there has been a notable progression in the breadth and depth of research topics. The shift towards longitudinal studies and program evaluations reflects a growing recognition of the need to assess the long-term effectiveness of dietary interventions in promoting oral health. The thematic evolution analysis highlights the emergence of interdisciplinary perspectives, wherein research integrates insights from fields, such as dentistry, public health, nutrition, and psychology. This interdisciplinary approach is crucial for comprehensively understanding the complex interplay between dietary habits, socio-behavioral determinants, and oral health outcomes.

Several factors influence the distribution of scientific research output across countries. Investment in research and development, like in the United States, Germany, and Japan, leads to higher publication rates due to robust infrastructure and support systems. Top-tier universities and industry collaborations attract talent and foster groundbreaking research, boosting output. Government policies, such as funding allocation and research incentives, are crucial. Cultural emphasis on academic excellence and economic stability also plays roles. International collaborations enhance output through shared resources and expertise. Disparities in education and opportunities within countries contribute to variations in research output across regions.

**Figure 14:**
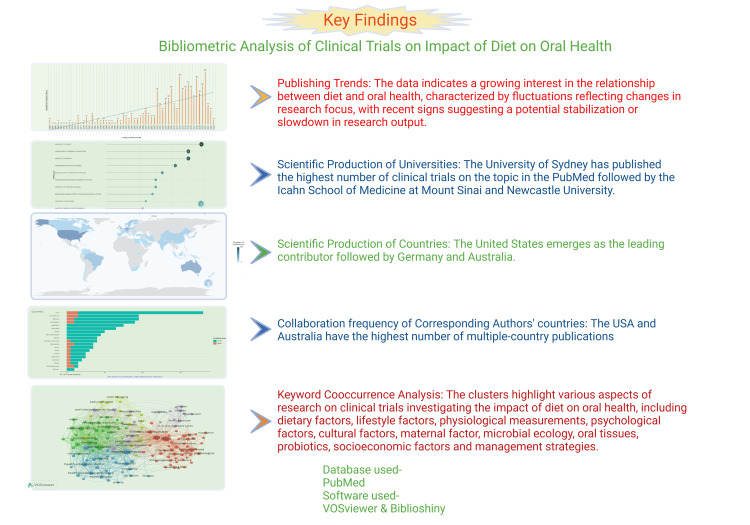
The key findings of a bibliometric analysis of clinical trials on the impact of diet on oral health. This figure is generated using the premium version of the Biorender App [22] with the agreement number DA26Q0O3WD. Note: This image is the author's own creation.

This study marks the pioneering effort in conducting a comprehensive bibliometric analysis coupled with a thematic examination of clinical trials exploring the impact of diet on oral health. To date, no such analysis has been undertaken in this domain, emphasizing the novelty and significance of this research endeavor. By amalgamating quantitative bibliometric analysis with qualitative thematic analysis, this study aims to elucidate trends, key contributors, and thematic patterns within the existing body of literature. Such an approach holds promise in unveiling the landscape of research interest, identifying knowledge gaps, and informing future research directions in the realm of diet-related oral health outcomes. Moreover, the findings of this study have the potential to guide evidence-based interventions and clinical practices geared toward promoting oral health and preventing oral diseases through dietary modifications. We have summarized the key findings in Figure 14.

While this bibliometric analysis provides valuable insights into the research landscape surrounding the impact of diet on oral health, it is essential to acknowledge several limitations. One limitation of this bibliometric analysis conducted on the impact of diet on oral health using PubMed data is the potential bias inherent in the database selection. PubMed primarily indexes biomedical literature, which may not fully represent research conducted in other disciplines related to oral health and nutrition. This could lead to a skewed portrayal of research trends and thematic clusters, particularly if relevant studies are published in journals not indexed by PubMed. Also, the data from PubMed doesn’t allow citation analysis. Additionally, while bibliometric analysis provides valuable insights into publication trends, collaboration networks, and geographic distribution of research output, it does not directly assess the quality or impact of individual studies. The quantity of publications alone may not accurately reflect the significance or rigor of the research conducted in this field.

However, the strength of this study lies in its integration of bibliometric analysis with thematic analysis. By combining these approaches, researchers can not only identify publication trends and key contributors but also gain a deeper understanding of the thematic landscape and evolving research priorities within the field of diet and oral health. Thematic analysis adds qualitative insights that complement the quantitative data obtained from bibliometric analysis, providing a more comprehensive picture of the research landscape and helping to uncover emerging themes, gaps, and interdisciplinary connections.

While there has been considerable research on the relationship between diet and oral health, several areas remain ripe for exploration and could benefit from future studies. These include conducting longitudinal studies to assess the sustained impact of dietary interventions on oral health outcomes over time, investigating the effectiveness of targeted dietary interventions tailored to specific populations such as pregnant women or individuals with chronic medical conditions, exploring the influence of emerging dietary trends like plant-based diets or ketogenic diets on oral health parameters, examining the interplay between psychological factors and dietary habits in shaping oral health behaviors, and conducting cross-cultural studies to understand how cultural and geographical factors impact dietary habits and oral health outcomes globally. By addressing these gaps in research, we can enhance our understanding of the complex relationship between diet and oral health and develop evidence-based strategies to promote long-term oral health and well-being.

## Conclusions

The analysis of clinical trial publications on the impact of diet on oral health over the past six decades reveals fluctuating trends, with notable peaks in 2021 followed by a recent decline, suggesting a general increasing interest in the topic but with indications of potential stabilization in research output. Primary contributors like Devizio and Proskin, along with leading institutions such as the University of Sydney, followed by Newcastle University and Icahn School of Medicine at Mount Sinai, continue to enrich research informing clinical practice and public health interventions worldwide. Nutrients have been identified as the most contributing source followed by Caries Research. The identified thematic clusters highlight diverse research facets, emphasizing the multidimensional nature of studying diet's impact on oral health. From physiological measurements to biochemical and microbiological aspects, research priorities have evolved dynamically, integrating interdisciplinary perspectives crucial for understanding complex interplays between dietary habits, demographics, and oral health outcomes. While early research emphasized experimental approaches and clinical trials, recent trends highlight a holistic understanding that incorporates longitudinal studies, behavioral determinants, and the exploration of physiological mechanisms. Emerging themes such as saliva chemistry, microbiota, and quality of life indicate evolving research priorities toward a comprehensive exploration of the relationship between diet, oral health, and overall well-being. The United States, Germany, and Australia stand out as leading contributors to scientific research globally, making notable contributions to the field. However, despite representation, larger nations such as China and India exhibit comparatively lower frequencies, indicating potential avenues for deeper investigation. Furthermore, variations in collaboration patterns across countries shape the global impact of their scientific endeavors. These findings collectively provide insights into the evolving landscape of research on diet and oral health, guiding future endeavors in this critical domain.
